# Ulcère Perforé-Bouché: A Case Report

**DOI:** 10.1016/j.gastha.2022.05.003

**Published:** 2022-05-14

**Authors:** Navkiran Randhawa, Toral Shastri, Misha Shah, Alex Yarbrough

**Affiliations:** 1 Franciscan Health Olympia Fields, Olympia Fields, Illinois; 2 Midwestern University Chicago College of Osteopathic Medicine, Downers Grove, Illinois

**Keywords:** Peptic Ulcer Disease, Endoscopy, Perforation, Ulcère Perforé-Bouché

## Abstract

Peptic ulcer disease refers to a break in the gastric or duodenal mucosal wall extending into the muscular mucosa. Although peptic ulcer disease commonly presents with dyspepsia, about 70% of patients initially present asymptomatically. A perforated peptic ulcer is a life-threatening complication of peptic ulcer disease that has high morbidity and mortality and requires emergent surgery. To prevent complications of peptic ulcer disease, an extensive history, physical examination, and appropriate imaging are required for appropriate management. In addition, the use of appropriate imaging and diagnostic modalities, such as an oral contrast computerized tomography of the abdomen, may lead to emergent treatment if complications arise. We present a unique case of a contained perforated duodenal ulcer within a fistula tract (Ulcère Perforé-Bouché) and diagnostic tools yielding detection and treatment of an Ulcère Perforé-Bouché. Abdominal x-rays may be inadequate the detect Ulcère Perforé-Bouché. However, an oral contract computerized tomography of the abdomen may have greater detection capabilities to diagnose cases of Ulcère Perforé-Bouché.

## Introduction

Peptic ulcer disease (PUD) refers to a break in the gastric or duodenal wall extending into the muscular mucosa.^1^ It results from an imbalance between protective factors allowing maintenance of the mucosa and factors causing damage to it.^1^ Common risk factors of PUD include *Helicobacter pylori* infection, chronic nonsteroidal anti-inflammatory drugs usage, gastric bypass surgery, cigarette smoking, and others.^1^ Per a systematic meta-analysis reviewed with data from the United States, the United Kingdom, and Europe, about 1–2 per 1000 people are affected annually by PUD.^2^ While the incidence is declining, complications from PUD have not similarly declined because of an aging population and frequent ulcerogenic medication usage.^2^

Patients with gastric and duodenal ulcers may present with retrosternal pain, early satiety, nausea, bloating, postprandial distress, and melena.^1^ Patients may also be asymptomatic and diagnosed with PUD once complications arise.^1^ Direct visualization of the ulcer on upper endoscopy is required for definitive diagnosis.^3^ Untreated ulcers can lead to bleeding, perforation, penetration to surrounding organs, and obstruction from fibrotic stricturing.^1^ Because of the high mortality associated with these complications (23.5% of patients with perforation die within 30 days for example), immediate diagnosis and surgical intervention are required for emergency treatment.^1^

We report a rare presentation of a contained peptic ulcer (Ulcère Perforé-Bouché), a complication of PUD. Secondary to a peptic ulcer, perforation typically presents with the sudden onset of acute severe abdominal pain.^1^ While our patient presented with worsening epigastric pain, she did not experience an acute abdomen or signs of active bleeding.

## Case Report

A 59-year-old female with a history of chronic obstructive pulmonary disease, bronchitis, and arthritis treated with nonsteroidal anti-inflammatory drugs was admitted for worsening epigastric abdominal pain and nausea that began 2 days before admission. She had a history of cholecystectomy performed 15 years earlier. The pain was nonradiating and rated as 4/10 on admission. Her symptoms were associated with melanic bowel movements and started after the onset of abdominal pain.

**Figure 1:**
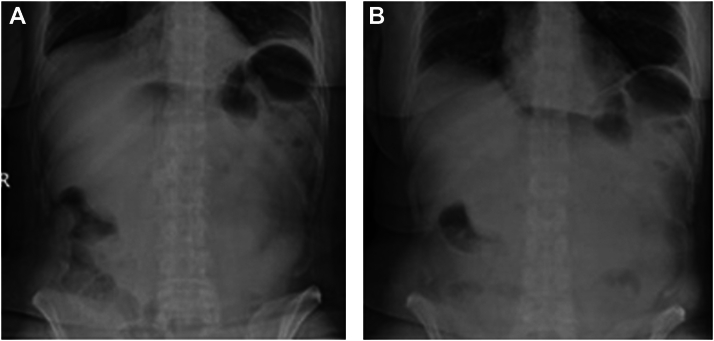
(A) First normal abdominal X-ray before EGD. (B) Second normal abdominal X-ray before EGD.

**Figure 2:**
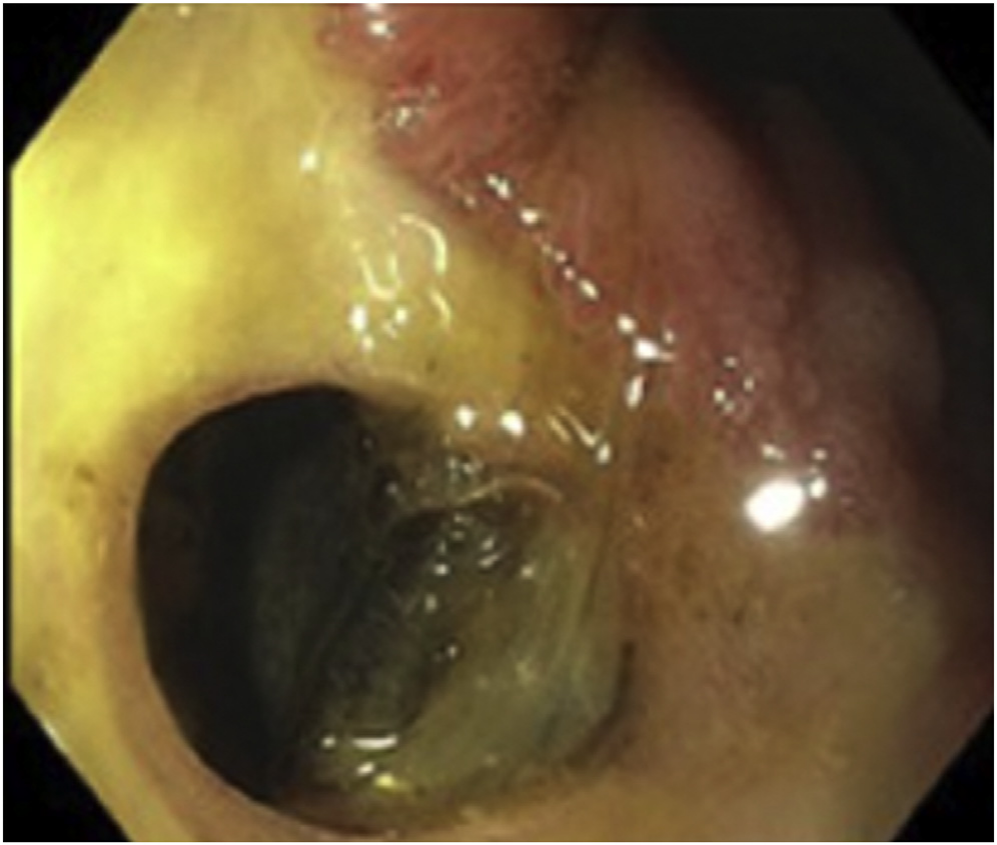
Endoscopic image of a contained perforated duodenal ulcer.

**Figure 3:**
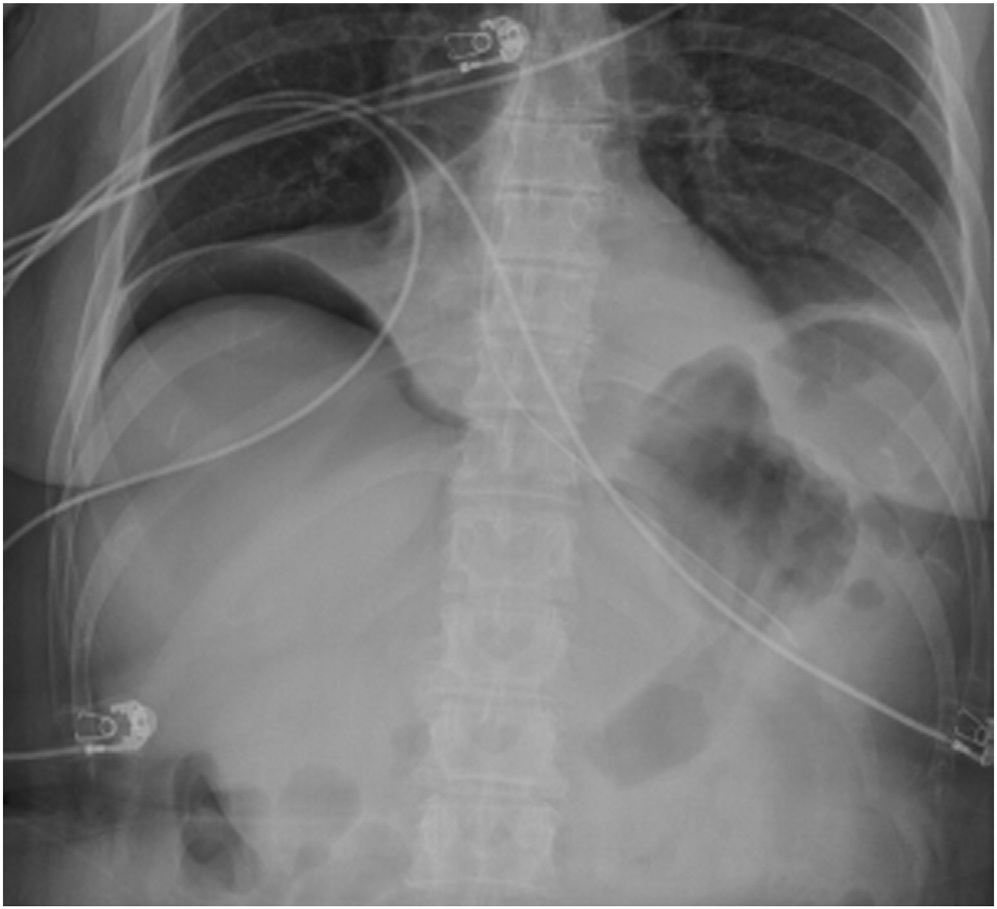
Abdominal X-ray portraying pneumoperitoneum after EGD.

Physical examination revealed evidence of prior laparoscopic abdominal surgery. She had mild tenderness to deep palpation in the epigastric region without guarding or rebound. The patient’s basic laboratory studies revealed a white blood cell count of 23.5 × 10^9^/L and lactic acid of 2.9 mmol/L. Her other laboratories were unremarkable. Computed tomography (CT) of the abdomen with intravenous contrast revealed haziness and nodularity of the anterior abdominal mesentery without extraluminal air. The abdominal x-rays obtained during admission and 1 hour before her upper esophagogastroduodenoscopy (EGD) revealed a nonspecific bowel gas pattern (Figure 1A and B). The EGD further revealed 1 nonbleeding duodenal ulcer covering half of the duodenal bulb circumference with a cratered area representing a fistula tract, also known as Ulcère Perforé-Bouché (Figure 2). A stat abdominal x-ray after the EGD revealed pneumoperitoneum (Figure 3).

The surgical team was consulted, and the patient was taken to the operating room immediately, and an exploratory laparotomy with an abdominal washout was performed. Dissection was carried out through the fascia with irrigation being used for better visualization. An opening was appreciated at the duodenal bulb. Omentum and falciform were placed over the perforation and tied down, creating an omental patch.

## Discussion

Ulcère Perforé-Bouché occurs when a perforated ulcer is directly blocked by an adjacent organ (liver, pancreas, spleen, etc), allowing containment of the perforation.^4^ An Ulcère Perforé-Bouché may be discovered incidentally during endoscopy.^4^ Typical symptoms, such as peritonitis or severe gastrointestinal (GI) hemorrhage, do not occur due to the containment.^4^ Our patient’s presentation of worsening abdominal pain for 2 days most likely represented a slow upper GI bleed.^5^ The lack of peritoneal signs and the abdominal free area made a perforated GI ulcer less likely.^5^ However, the perforation site was contained by the patient’s overlapping omentum and revealed during EGD, causing the patient’s atypical presentation.

When a patient presents with acute abdominal pain, it is important to consider causes, such as appendicitis, cholecystitis, bowel obstruction, urinary colic, perforated peptic ulcer, pancreatitis, etc.^6^ Despite significant diagnostic limitations, initial radiological evaluation includes a plain abdominal radiograph.^6^ The initial and pre-EGD abdominal x-rays did not have any significant findings in our patient's case. We contemplate whether an oral contrast CT of the abdomen would have helped detect the contained perforated ulcer.

Gas insufflation is regularly used for optimal visualization during GI endoscopy with a constant rate of 1.4 L/min.^7^ However, suspicion for Ulcère Perforé-Bouché may indicate a decreased rate of insufflation to prevent the worsening of the perforation. Although further studies are indicated to determine the appropriate rate of insufflation to avoid complications, physicians must be aware of this possibility. Patients with worsening perforations may present within 12 hours of the procedure with pain, fever, abdominal distension, nausea, and peritoneal signs.^8^

Timely diagnosis and emergent surgical intervention of a perforated PUD are essential to improve prognosis. This case reports a rare finding of a contained perforated ulcer while also emphasizing the importance of careful endoscopic gas insufflation during diagnostic endoscopy. Significant insufflation in Ulcère Perforé-Bouché can worsen perforation. This case also explores the use of oral contrast CT abdomen vs repeat abdominal x-ray in patients with a suspected perforated bowel. The multiple abdominal x-rays completed during the patient’s hospital course were unable to detect the Ulcère Perforé-Bouché. This raises the question of whether an oral contrast CT abdomen could be used to better diagnose cases of Ulcère Perforé-Bouché.
